# Factors associated with dental service use in Brazil according to Andersen's Behavioral Model: analysis of the 2023 Brazilian National Oral Health Survey

**DOI:** 10.1590/1980-549720260016.supl.1

**Published:** 2026-07-10

**Authors:** Debora Lana Alves Monteiro, Juliana Pereira da Silva Faquim, Rênnis Oliveira da Silva, Edson Hilan Gomes de Lucena, Yuri Wanderley Cavalcanti

**Affiliations:** I Universidade Federal da Paraiba, Programa de Pós-Graduação em Odontologia – João Pessoa (PB), Brazil.; II University of Saskatchewan, College of Dentistry – Saskatoon, Saskatchewan, Canadá.; III Universidade Federal da Pernambuco, Programa de Pós-Graduação em Saúde da Criança e do Adolescente – Recife (PE), Brazil.; IV Universidade Federal da Paraiba, Departamento de Odontologia Clínica e Social – João Pessoa (PB), Brazil.

**Keywords:** Universal health coverage, Health status disparities, Socioeconomic factors, Oral health

## Abstract

**Objective:**

To analyze factors associated with the time since the last dental visit among Brazilians aged 15–19, 35–44, and 65–74 years, according to Andersen's Behavioral Model.

**Methods:**

This cross-sectional analytical study used microdata from a nationally representative oral health survey. Data from 21,385 individuals were analyzed. The outcome was the time since the last dental visit (≤1 year vs. >1 year). The independent variables were grouped into predisposing, facilitating, and perceived need factors according to Andersen's Behavioral Model. Analyses accounted for the complex sampling design and were performed using multilevel logistic regression with random intercepts at the municipality level, estimating odds ratios (OR) and 95% confidence intervals (95%CI).

**Results:**

Older adults (OR 1.436; 95%CI 1.242–1.660), Black (OR 1.161; 95%CI 1.011–1.334) and Brown individuals (OR 1.096; 95%CI 1.005–1.196) were more likely to report a dental visit more than one year prior. Higher education was strongly associated with lower odds of delayed visits (OR 0.383; 95%CI 0.318–0.461). Poor self-rated oral health (OR 2.486; 95%CI 2.041–3.028) increased the likelihood of delayed visits, whereas dental pain was associated with more recent use (OR 0.413; 95%CI 0.382–0.448). Use of private services was associated with lower odds of delayed visits (OR 0.820; 95%CI 0.732–0.919). Municipal coverage and the presence of specialized centers were not significantly associated.

**Conclusion:**

Sociodemographic characteristics and perceived need factors remain central determinants of recent dental service utilization patterns in Brazil.

## INTRODUCTION

Regular use of dental services is an important indicator of access and equity in oral health^1^, reflecting not only the availability of care resources, but also the social, economic, and cultural conditions that influence care-seeking behavior^1–3^. Patterns of dental service utilization, including time since the last dental visit, are widely examined in health services research as measures of realized access and inequalities in care^1,2^.

Evidence consistently shows that the use of dental services remains unequal across population groups, influenced by factors such as income level, education level, ethnicity, and insurance coverage^1,2^. These inequalities persist even in countries with well-structured health systems, indicating that not only structural, but also behavioral and contextual barriers affect effective access^3^.

In Brazil, although the Unified Health System (Sistema Único de Saúde – SUS) has expanded the provision of dental services, the effectiveness of this expansion has been uneven^4,5^. Organizational barriers, such as a lack of human resources and inadequate infrastructure, and geographic factors, such as distance to services, continue to limit access, especially in poorer regions^6^.

In a study conducted by Fagundes et al.^7^ using data from the 2019 National Health Survey (Pesquisa Nacional de Saúde – PNS), it was found that 53.2% of adults and 34.3% of older adults had used dental services in the 12 months prior to the interview. Comparing with data from the 2013 PNS, the authors themselves highlighted a slight increase in use, from 47.7 to 53.2% among adults and from 29.4 to 34.3% among older adults, suggesting modest advances in access and the persistence of socioeconomic and regional inequalities. Furthermore, having a dental plan is more common among White individuals, residents of urban areas, and those with a higher level of education, highlighting the influence of social and economic factors on access to oral health^7,8^.

Recent reviews^1,9,10^ reinforce that the use of dental services is also strongly associated with the perception of need, attitudes towards oral health, and the sociocultural context of individuals, requiring studies with analytical approaches that consider multiple determinants and levels of influence. In this regard, Andersen's Behavioral Model^11^ provides a robust theoretical structure for examining health service utilization. Originally proposed by Andersen and Newman^12^ and later reformulated by Andersen^11^, the model postulates that the use of services is the result of the interaction between three groups of determinants: predisposing factors (demographic and social characteristics), enabling factors (individual and contextual resources that facilitate access), and need factors (perceived or objectively evaluated health conditions).

This framework has been consistently applied in both national^13–15^ and international^16–18^ research to investigate inequalities in access to dental care and to assess the influence of individual and contextual determinants on service utilization. In Brazil, multilevel analyses grounded in Andersen's model have demonstrated that both socioeconomic characteristics and contextual municipal factors shape the use of public dental services^13–15^.

Although national surveys such as the PNS have substantially contributed to documenting inequalities in dental service use^7^, SB Brasil 2023^19^ provides a nationally representative dataset specifically designed to assess oral health conditions and patterns of service utilization, enabling a more comprehensive and oral health-focused evaluation of recent determinants. Moreover, despite substantial investments and expansion of primary oral health care within the SUS over the past two decades^4^, inequalities and barriers to access remain^5,6^. It therefore remains unclear whether these structural advances have translated into meaningful reductions in inequalities in recent dental service utilization.

In this context, the present study aims to analyze the factors associated with recent dental service utilization, operationalized as time since the last dental visit among Brazilians aged 15 years or older, based on Andersen's Behavioral Model, and to examine whether sociodemographic, perceptual, and contextual determinants remain associated with inequalities in recent dental service utilization.

## METHODS

This is a cross-sectional, population-based study with a descriptive and analytical design, based on data from the National Oral Health Survey (SB Brasil 2023). Data collection was conducted between 2022 and 2023, following nationally standardized protocols. The sampling plan considered 53 geographic domains (the 26 states, the Federal District, and state capitals) and used probability sampling stratified by clusters (census tracts)^19,20^. SB Brasil 2023 is a nationwide household-based survey designed to assess oral health conditions and service utilization in Brazil. The sampling followed a complex, multistage probabilistic design. Within each geographic domain, municipalities were selected, followed by a random selection of census tracts and households. All eligible individuals within the predefined age groups of 15 to 19 years, 35 to 44 years, and 65 to 74 years were invited to participate. Data were collected by trained and calibrated dental examiners and interviewers, according to procedures described in the official survey manual^19,20^.

The initial sample comprised 25,372 participants aged 15 years or older, of whom 21,385 were included in the analyses after excluding individuals with missing information in the dependent variable or in key independent variables. A complete-case analysis approach was adopted to ensure consistency in multivariable modeling. The main exclusions were related to missing responses in self-reported measures and skin color.

Data extracted from SB Brasil 2023 were as follows: sex (male or female), age group (15–19 years, 35–44 years, 65–74 years), self-reported skin color (White, Black, Brown), years of education (no education, 1–4 years, 5–8 years, 9 years or more), self-rated oral health (very good, good, fair, poor, and very poor), self-rated need for treatment (yes or no), self-reported toothache (yes or no), self-reported facial pain (yes or no), and impact of oral health on quality of life (yes or no), and location of dental service use (public or private sector).

According to Andersen's Behavioral Model, the independent variables were conceptually organized into three domains. Predisposing factors included sex, age group, self-reported skin color, and years of education. Enabling factors included the type of dental service used, municipal oral health coverage in primary care, and availability of Dental Specialty Centers (Centro de Especialidades Odontológicas — CEO) in the municipality. Need factors comprised self-rated oral health, perceived need for treatment, self-reported toothache, self-reported facial pain, and the impact of oral health on daily activities.

Skin color was self-reported by the participant at the time of the interview. Participants who did not provide their skin color, as well as those who reported Asian or Indigenous skin color, were removed from the sample due to low case frequency. The impact of oral health on quality of life was assessed using the Oral Impact questionnaire on Daily Performance (OIDP), consisting of nine questions that asked about the perception of how oral health influences activities of daily living. Participants who answered "no" to all questions were considered to have no impact on their oral health quality of life. Self-reported toothache and facial pain referred to the presence of symptoms within the six months prior to the interview, according to the survey questionnaire.

In addition to individual-level variables, contextual municipal data were collected on oral health coverage in primary health care and the availability of CEOs in the municipality. CEOs are public secondary care facilities within the Brazilian SUS that provide specialized dental procedures, including endodontics, periodontics, oral surgery, and care for patients with special needs. Municipal oral health coverage in primary care was defined as the proportion of the population covered by Oral Health Teams within the Family Health Strategy and was obtained from official administrative data available on the "e-Gestor Atenção Primária à Saúde" platform for the year 2023. Coverage was categorized as up to 80% and above 80%. Data on health service availability were extracted from public reports on the "e-Gestor Atenção Primária à Saúde" platform (https://relatorioaps.saude.gov.br/) for the year 2023.

The dependent variable was recent dental service utilization, operationalized as time elapsed since the last dental visit, categorized as up to one year and more than one year ago. Individuals who reported never having visited a dentist were excluded from the analysis.

The data were imported and analyzed using Stata software (version 19). Weighted estimates were obtained based on the complex study design, according to guidelines from the database provided by the Ministry of Health^20^.

Multilevel logistic regression models with random intercepts for municipalities were fitted. The fixed effects were introduced according to the conceptual domains of Andersen's model, including predisposing, enabling, and need factors, as previously described.

**Figure 1:**
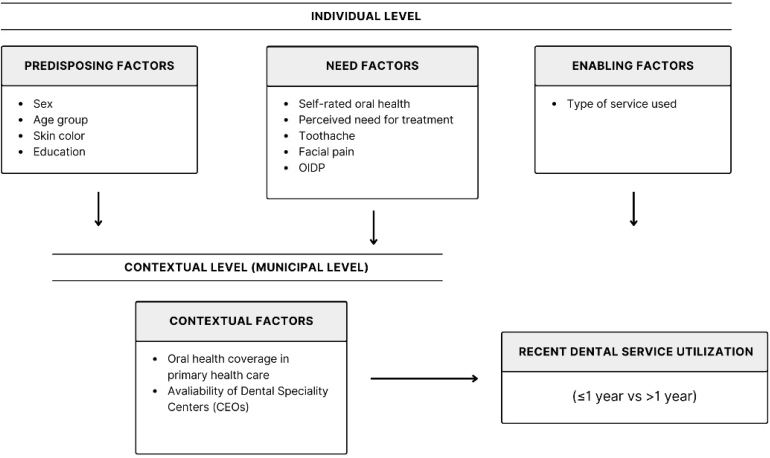
Conceptual framework of factors associated with recent dental service utilization according to Andersen's Behavioral Model, SB Brasil 2023.

Thus, a hierarchical multilevel model was constructed to estimate the cumulative effect of individual and contextual determinants on recent dental service utilization. Figure 1 illustrates the conceptual framework adopted in this study, based on Andersen's Behavioral Model, and the organization of individual and contextual variables included in the analysis.

Model fit was assessed using the Akaike Information Criterion (AIC) and the Bayesian Information Criterion (BIC), with lower values indicating better fit. The proportion of variance attributable to the municipal level was estimated using the intraclass correlation coefficient (ICC). The final model, which considered the effects of sociodemographic, self-reported, and service variables, was found to be a significantly good fit.

The effect of the independent variables was estimated using odds ratios (OR) using a robust estimator. Variables with a p<0.05 were considered statistically significant, considering a 95% confidence interval.

### Data availability statement:

The entire dataset supporting the results of this study is available upon request from the corresponding author. The dataset is not publicly available due to database integration performed by the authors.

## RESULTS

The study sample comprised 25,372 participants. Of this total, 21,385 observations were analyzed, as some participants had missing data. The weighted frequencies of the study variables are presented in Table 1.

**Table 1:** Weighted frequencies of the variables under study and 95% confidence interval, considering the complex sampling design of the study.

		Weighted frequency (%)	95%CI	
How long have you been using dental services?				
	Up to 1 year	51.89	48.99	54.76
	Over a year ago	48.11	45.24	51.01
Sex				
	Masculine	38.76	37.13	40.42
	Feminine	61.24	59.58	62.87
Age range (years old)				
	15 to 19	23.59	21.91	25.36
	35 to 44	55.60	53.37	57.80
	65 to 74	20.81	19.23	22.49
Skin color				
	White	44.63	41.03	48.28
	Black	12.64	10.99	14.49
	Brown	42.73	39.35	46.19
Education (years of study)				
	None	2.95	2.40	3.62
	1 to 4	8.15	6.88	9.62
	5 to 8	14.71	13.19	16.38
	9 or older	74.19	71.52	76.69
Oral health self-assessment				
	Very good	8.12	7.09	9.29
	Good	46.01	43.81	48.23
	Regular	33.43	31.59	35.32
	Bad	9.47	8.15	10.96
	Very bad	2.98	2.29	3.86
Self-assessment of treatment need				
	No	31.35	28.41	34.44
	Yes	68.65	65.56	71.59
Has a toothache				
	No	79.19	77.05	81.17
	Yes	20.81	18.83	22.95
Has pain in the face				
	No	83.15	81.03	85.07
	Yes	16.85	14.93	18.97
Impact of oral health on quality of life				
	No impact	51.27	47.77	54.77
	With impact	48.73	45.23	52.23
Place of use of dental services				
	Public	51.09	47.62	54.55
	Private	48.91	45.45	52.38
Oral Health Coverage in PHC				
	Up to 80%	71.81	65.99	76.97
	More than 80%	28.19	23.03	34.01
Has CEO				
	No	28.64	20.89	37.88
	Yes	71.36	62.12	79.11

C: confidence interval; PHC: Primary Health Care; CEO: Centro de Especialidades Odontológicas.

Of the total number of individuals included in the analysis, 48.11% (95%CI 45.24–51.01) reported having used a dental service for more than a year. Participants were mostly female (61.24%; 95%CI 59.58–62.87), aged 35 to 44 (55.60%; 95%CI 53.37–57.80), and self-identified as White (44.63%; 95%CI 41.03–48.28), with nine or more years of education (74.19%; 95%CI 71.52–76.69). Most participants self-rated their oral health as "good" (46.01%; 95%CI 43.81–48.23), and 68.65% (95%CI 65.56–71.59) reported perceived need for dental treatment. Toothache (20.81%; 95%CI 18.83–22.95) and facial pain (16.85%; 95%CI 14.93–18.97) were less frequently reported, and the percentage of individuals reporting an impact from oral health on their quality of life was balanced. Participants reported using public services in 51.09% (95%CI 47.62–54.55) of cases, residing in cities with oral health coverage below 80%, and with the presence of a CEO.

Multilevel logistic regression results are shown in Table 2. Sequential models were fitted according to Andersen's conceptual framework. In Model 1 (Predisposing factors), which included only sociodemographic variables, older age and lower educational attainment were strongly associated with delayed dental utilization. Individuals aged 65–74 years had significantly higher odds of having their last dental visit more than one year ago (OR 1.716; 95%CI 1.477–1.995). A clear educational gradient was observed, with individuals with nine or more years of schooling presenting substantially lower odds of delayed utilization (OR 0.381; 95%CI 0.319–0.455). Black and Brown individuals also showed higher odds compared to White ones. After inclusion of patient-reported need variables in Model 2, some associations were attenuated but remained statistically significant, indicating partial mediation by perceived oral health conditions. Self-rated oral health showed the strongest associations in the model. Individuals rating their oral health as "very bad" presented markedly higher odds of delayed utilization (OR 2.821; 95%CI 2.277–3.494). Perceived need for treatment remained associated (OR 1.206; 95%CI 1.084–1.341). Conversely, participants reporting toothache were less likely to have delayed dental visits (OR 0.419; 95%CI 0.386–0.454), suggesting symptom-driven service utilization. In the fully adjusted model (Model 3), which incorporated enabling factors, most sociodemographic and need variables remained associated with delayed utilization. Female sex was associated with lower odds of delayed dental visits compared to males (OR 0.799; 95%CI 0.753–0.848). Individuals aged 65–74 years continued to present elevated odds (OR 1.436; 95%CI 1.242–1.660), although with attenuation compared to Model 1. Racial disparities persisted, with Black (OR 1.161; 95%CI 1.011–1.334) and Brown individuals (OR 1.096; 95%CI 1.005–1.196) showing higher odds of delayed utilization. Education remained strongly associated. Participants with nine or more years of schooling had substantially lower odds of delayed dental visits (OR 0.383; 95%CI 0.318–0.461), reinforcing the magnitude of the educational gradient.

**Table 2:** Multiple Multilevel Logistic Regression Model that estimated the factors associated with using dental services for more than one year, considering a hierarchical model of variables related to socioeconomic factors, patient-reported measures, and service availability.

		Model 1 (Individual sociodemographics)			Model 2 (Sociodemographic+PROM)			Model 3 (Sociodemographic+PROM+Service)		
		OR	95%CI		OR	95%CI		OR	95%CI	
Sex (male)		ref.			ref.			ref.		
	Feminine	0.778	0.735	0.823	0.792	0.747	0.839	0.799	0.753	0.848
Age range (15 to 19 years)		ref.			ref.			ref.		
	35 to 44 years old	1.191	1.063	1.336	1.099	0.985	1.226	1.115	0.998	1.245
	65 to 74 years old	1.716	1.477	1.995	1.399	1.212	1.615	1.436	1.242	1.660
Skin color (white)		ref.			ref.			ref.		
	Black	1.217	1.075	1.378	1.193	1.045	1.362	1.161	1.011	1.334
	Brown	1.122	1.025	1.229	1.105	1.013	1.205	1.096	1.005	1.196
Education (did not study)		ref.			ref.			ref.		
	1 to 4 years	0.797	0.649	0.980	0.799	0.643	0.992	0.824	0.657	1.032
	5 to 8 years old	0.633	0.533	0.751	0.620	0.521	0.737	0.631	0.527	0.756
	9 years or older	0.381	0.319	0.455	0.367	0.307	0.440	0.383	0.318	0.461
Oral health self-assessment (very good)					ref.			ref.		
	Good				1.267	1.123	1.429	1.250	1.105	1.415
	Regular				1.810	1.562	2.098	1.769	1.523	2.055
	Bad				2.552	2.116	3.079	2.486	2.041	3.028
	Very bad				2.821	2.277	3.494	2.739	2.201	3.408
Self-assessment of need for treatment (no)					ref.			ref.		
	Yes				1.206	1.084	1.341	1.202	1.079	1.338
Toothache (no)					ref.			ref.		
	Yes				0.419	0.386	0.454	0.413	0.382	0.448
Pain in the face (no)					ref.			ref.		
	Yes				0.936	0.842	1.040	0.939	0.843	1.045
Impact of oral health on quality of life (no)					ref.			ref.		
	Yes				0.829	0.754	0.912	0.830	0.754	0.914
Type of service used (public)								ref.		
	Private							0.820	0.732	0.919
Oral health coverage in PHC (up to 80%)								ref.		
	Above 80%							0.904	0.750	1.090
CEO presence (no)								ref.		
	Yes							1.084	0.891	1.320
	**Null model**	**Model 1**			**Model 2**			**Model 3**		
ICC	9.05%	8.74%			8.07%			8.20%		
AIC	33580.53	31124.33			28018.19			27502.59		
BIC	33596.75	31205.07			28138.01			27646.06		

PROM: participant self-reported outcomes; OR: odds ratio; CI: confidence interval; ref.: reference category; PHC: Primary Health Care; ICC: intraclass correlation coefficient; AIC: Akaike Information Criterion; BIC: Bayesian Information Criterion.

Need factors continued to demonstrate the largest effect sizes. Individuals rating their oral health as "very bad" had more than threefold higher odds of delayed utilization (OR 2.739; 95%CI 2.201–3.408), representing the strongest association observed in the model. Perceived need for treatment remained associated (OR 1.202; 95%CI 1.079–1.338). Toothache remained inversely associated (OR 0.413; 95%CI 0.382–0.448).

Regarding enabling factors, use of private dental services was associated with lower odds of delayed utilization (OR 0.820; 95%CI 0.732–0.919). However, municipal oral health coverage above 80% (OR 0.904; 95%CI 0.750–1.090) and the presence of a CEO (OR 1.084; 95%CI 0.891–1.320) were not significantly associated.

Additionally, the ICC decreased from 9.05% in the null model to 8.20% in the fully adjusted model, indicating modest, yet persistent, contextual variability among municipalities.

## DISCUSSION

The results of this study indicate that sociodemographic characteristics and self-reported need-related measures are strongly associated with recent dental service utilization in Brazil. Although municipal oral health coverage and the availability of CEOs were not statistically associated with time since the last visit, individuals who reported using private services were more likely to have had a dental visit within the previous year.

Similar results were observed in national studies^7,8,21^ that also identified inequalities in the use of dental services according to the type of service, with a consistent advantage for private sector users. These studies indicate that having a health plan is associated with a higher frequency of dental appointments and regular use of services, while supplementary access remains concentrated among individuals with higher income and education^8,21^. Furthermore, public system users are less likely to use dental services regularly and have a longer time since their last appointment, highlighting the persistence of structural inequities in in oral health care utilization in Brazil^7^.

The present findings reinforce that sociodemographic factors such as skin color, education level, and age remain important determinants of recent dental service utilization in Brazil, consistent with previous studies^5–7,13^. A clear educational gradient was observed, with higher schooling showing a strong protective association against delayed dental visits, suggesting persistent social patterning in oral health service use.

Need-related factors demonstrated the strongest associations in the fully adjusted model. Individuals who rated their oral health as poor or very poor showed substantially higher odds of reporting a dental visit more than one year prior, while those reporting a toothache were more likely to have had a recent visit, indicating symptom-driven patterns of utilization. These findings align with literature emphasizing the central role of perceived need in health service use^1,2,13^ and reinforce the explanatory capacity of Andersen's model when applied to oral health contexts.

The strong associations observed for self-reported measures, particularly self-rated oral health and perceived need for treatment, suggest that patient-reported indicators are closely related to recent patterns of dental service utilization. Although this study did not evaluate screening strategies, these findings indicate that self-reported measures may serve as relevant markers for identifying groups with different utilization profiles.

In this context, recent studies^22–24^ have highlighted the potential of digital technologies and teledentistry in expanding service reach, improving care quality, and monitoring perceived needs, especially within the SUS. Therefore, future research could explore how patient-reported indicators might be integrated into care planning and service organization strategies, especially for socially vulnerable populations.

Regarding contextual enabling factors, no statistically significant association was observed between municipal oral health coverage or the presence of a CEO and recent utilization. To some extent, the expansion of dental services in Brazil over the last 20 years^25–28^ has contributed to reducing inequities in this regard. However, these administrative indicators may not fully capture dimensions such as geographic accessibility, service organization, waiting times, or effective service availability. Therefore, the absence of an association should be interpreted cautiously and does not necessarily imply that contextual factors are irrelevant, but rather that the measures available may not encompass all relevant dimensions of enabling resources.

Interestingly, even considering only individuals who reported using public services, no effect of oral health coverage or the presence of a CEO (data not shown) was observed. This suggests that structural service indicators alone may not explain differences in utilization intervals, and may mean that that individual-level characteristics and perceived need remain central determinants^1,2,5,7,13^.

It is important to emphasize that this study evaluated time since the last dental visit among individuals who had previously accessed services. Therefore, the findings should not be interpreted as direct measures of access barriers or difficulty in obtaining care. Differences observed may reflect variations in need, preferences, health beliefs, service organization, or care models, and causal mechanisms cannot be inferred from the cross-sectional design.

As limitations, it can be noted that this study did not consider the effect of the income variable in the construction of predictive models. This is because the income variable has a non-response frequency of approximately 30%, which led the authors to omit this variable in the study to avoid losing the robustness of the sample analysis. Even so, it is recognized that income is an important social determinant of the use of dental services and that its absence may partially limit the understanding of economic inequalities associated with access.

Additionally, the outcome was based on self-reported information, which may be subject to recall bias. The cross-sectional nature of the study prevents temporal inference. Individuals who had never visited a dentist were excluded from the analysis, which limits conclusions about absolute access to services. Finally, contextual indicators such as primary care coverage and CEO presence represent administrative measures and may not fully reflect actual availability, quality, or accessibility of services at the local level.

Given these findings, the results reinforce the importance of approaches that consider the interaction between individual, social, and structural determinants of dental service utilization. About predisposing factors, the persistence of social and racial inequalities is consistent with literature emphasizing the relevance of intersectoral strategies aimed at reducing inequities and strengthening oral health literacy^2,29^.

Concerning enabling factors, strengthening Primary Oral Health Care and enhancing integration between Oral Health Teams and CEOs within the health care network are essential measures to overcome fragmented care, promote continuity between levels of care, and make the system more effective and equitable, reducing regional inequalities^30^.

Finally, regarding perceived need factors, the incorporation of self-reported indicators, such as Patient-Reported Outcome Measures and Patient-Reported Experience Measures, has been suggested as a strategy to improve needs monitoring and quality assessment, supporting more patient-centered approaches^31,32^.
