# Supplementary material for: Factors associated with dental service use in Brazil according to Andersen's Behavioral Model: analysis of the 2023 Brazilian National Oral Health Survey
Source: Rev Bras Epidemiol. 2026 Jul 10;29(Suppl 1):e260016supl1. doi: 10.1590/1980-549720260016.supl.1 (PMC13360980; doi:10.1590/1980-549720260016.supl.1)
Supplement: Translation

# Translation: Fatores associados ao uso de serviços odontológicos no Brasil segundo o Modelo Comportamental de Andersen: análise da Pesquisa Nacional de Saúde Bucal 2023

**DOI:** 10.1590/1980-549720260016.supl.1.2

**Authors:** Debora Lana Alves Monteiro, Juliana Pereira da Silva Faquim, Rênnis Oliveira da Silva, Edson Hilan Gomes de Lucena, Yuri Wanderley Cavalcanti

**Affiliations:** I Universidade Federal da Paraíba, Programa de Pós-Graduação em Odontologia – João Pessoa (PB), Brasil.; II University of Saskatchewan, College of Dentistry – Saskatoon, Saskatchewan, Canadá.; III Universidade Federal da Pernambuco, Programa de Pós-Graduação em Saúde da Criança e do Adolescente – Recife (PE), Brasil.; IV Universidade Federal da Paraíba, Departamento de Odontologia Clínica e Social – João Pessoa (PB), Brasil.

**Keywords:** Cobertura universal de saúde, Desigualdades em saúde, Fatores socioeconômicos, Saúde bucal

## Abstract

**Objetivo:**

Analisar os fatores associados ao tempo desde a última consulta odontológica entre brasileiros nas faixas etárias de 15–19, 35–44 e 65–74 anos, segundo o Modelo Comportamental de Andersen.

**Métodos:**

Estudo transversal analítico, que utilizou microdados de um inquérito nacional representativo de saúde bucal. Foram analisados dados de 21.385 indivíduos. O desfecho foi o tempo desde a última consulta odontológica (≤1 ano vs. >1 ano). As variáveis independentes foram agrupadas em fatores predisponentes, facilitadores e necessidade percebida, conforme o Modelo de Andersen. As análises consideraram o desenho amostral complexo e foram realizadas por meio de regressão logística multinível com intercepto aleatório no nível do município, estimando razões de chances (OR) e intervalos de confiança de 95% (IC95%).

**Resultados:**

Idosos (OR 1,436; IC95% 1,242–1,660), pretos (OR 1,161; IC95% 1,011–1,334) e pardos (OR 1,096; IC95% 1,005–1,196) apresentaram maior probabilidade de relatar que a última consulta ocorreu há mais de um ano. Maior escolaridade esteve fortemente associada a menores chances de utilização tardia (OR 0,383; IC95% 0,318–0,461). Autoavaliação negativa da saúde bucal (OR 2,486; IC95% 2,041–3,028) aumentou a probabilidade de consulta tardia, enquanto dor de dente esteve associada ao uso mais recente (OR 0,413; IC95% 0,382–0,448). O uso de serviços privados esteve associado a menores chances de utilização tardia (OR 0,820; IC95% 0,732–0,919). A cobertura municipal de saúde bucal e a presença de centros especializados não apresentaram associação significativa.

**Conclusão:**

Características sociodemográficas e fatores de necessidade percebida permanecem como determinantes centrais dos padrões recentes de utilização de serviços odontológicos no Brasil.

## INTRODUÇÃO

O uso regular de serviços odontológicos é um importante indicador de acesso e equidade em saúde bucal^1^, refletindo não só a disponibilidade de recursos assistenciais, mas também as condições sociais, econômicas e culturais que influenciam o comportamento de busca por cuidados^1–3^. Os padrões de utilização de serviços odontológicos, incluindo o tempo desde a última consulta odontológica, são amplamente investigados em pesquisas sobre serviços de saúde como medidas de acesso realizado e desigualdades na assistência^1,2^.

As evidências mostram consistentemente que o uso de serviços odontológicos permanece desigual entre os grupos populacionais, influenciado por fatores como renda, escolaridade, etnia e cobertura de plano de saúde^1,2^. Essas desigualdades persistem mesmo em países com sistemas de saúde bem estruturados, indicando que não só as barreiras estruturais, mas também as comportamentais e contextuais afetam o acesso efetivo^3^.

No Brasil, embora o Sistema Único de Saúde (SUS) tenha ampliado a oferta de serviços odontológicos, a eficácia dessa expansão tem sido desigual^4,5^. As barreiras organizacionais, como a falta de recursos humanos e infraestruturas inadequadas, e os fatores geográficos, como a distância dos serviços, continuam a limitar o acesso, especialmente em regiões mais pobres^6^.

Fagundes et al.^7^, utilizando dados da Pesquisa Nacional de Saúde (PNS) de 2019, verificaram que 53,2% dos adultos e 34,3% dos idosos utilizaram serviços odontológicos nos 12 meses anteriores à entrevista. Comparando com os dados da PNS de 2013, os autores destacaram um aumento discreto na utilização, de 47,7 para 53,2% entre adultos e de 29,4 para 34,3% entre idosos, sugerindo avanços modestos no acesso e na persistência de desigualdades socioeconômicas e regionais. Além disso, ter um plano odontológico é mais comum entre os brancos, moradores de áreas urbanas e aqueles com maior escolaridade, destacando a influência de fatores sociais e econômicos no acesso à saúde bucal^7,8^.

Revisões de literatura recentes^1,9,10^ reforçam que o uso de serviços odontológicos também está fortemente associado à percepção de necessidade, às atitudes em relação à saúde bucal e ao contexto sociocultural dos indivíduos, de modo que estudos com abordagens analíticas que considerem múltiplos determinantes e níveis de influência são necessários. A este respeito, o Modelo Comportamental de Andersen^11^ fornece uma estrutura teórica robusta para investigar a utilização de serviços de saúde. Originalmente proposto por Andersen e Newman^12^ e posteriormente reformulado por Andersen^11^, o modelo postula que o uso de serviços resulta da interação entre três grupos de determinantes: fatores predisponentes (caraterísticas sociodemográficas), facilitadores (recursos individuais e contextuais que facilitam o acesso), e necessidade percebida (condições de saúde percebidas ou objetivamente avaliadas).

Esse modelo tem sido consistentemente aplicado tanto em pesquisas nacionais^13–15^ quanto internacionais^16–18^ para investigar desigualdades no acesso aos cuidados odontológicos e para avaliar a influência de determinantes individuais e contextuais na utilização de serviços. No Brasil, análises multinível baseadas no modelo de Andersen demonstraram que tanto as caraterísticas socioeconômicas como os fatores contextuais municipais moldam a utilização de serviços públicos odontológicos^13–15^.

Embora pesquisas nacionais como a PNS tenham contribuído substancialmente para documentar as desigualdades na utilização de serviços odontológicos^7^, a pesquisa SB Brasil 2023^19^ fornece um conjunto de dados nacionalmente representativo e especificamente projetado para avaliar as condições de saúde bucal e os padrões de utilização dos serviços, permitindo uma avaliação mais abrangente dos determinantes recentes e com foco na saúde bucal. Além disso, apesar de investimentos substanciais e da expansão da Atenção Primária à Saúde Bucal no âmbito do SUS nas últimas duas décadas^4^, as desigualdades e barreiras ao acesso permanecem^5,6^. Portanto, não está claro se esses avanços estruturais resultaram em reduções significativas nas desigualdades na recente utilização de serviços odontológicos.

Nesse contexto, o presente estudo tem como objetivo analisar os fatores associados à recente utilização de serviços odontológicos, operacionalizada como tempo desde a última consulta odontológica entre brasileiros com 15 anos ou mais, com base no Modelo Comportamental de Andersen, e avaliar se os determinantes sociodemográficos, perceptivos e contextuais permanecem associados às desigualdades na recente utilização de serviços odontológicos.

## MÉTODOS

Estudo transversal, de base populacional, com desenho descritivo e analítico, com base em dados da Pesquisa Nacional de Saúde Bucal (SB Brasil 2023). A coleta de dados foi realizada entre 2022 e 2023, seguindo protocolos padronizados nacionalmente. O plano amostral considerou 53 domínios geográficos (os 26 estados, o Distrito Federal e as capitais) e utilizou amostragem probabilística estratificada por conglomerados (setores censitários)^19,20^. A SB Brasil 2023 é uma pesquisa nacional de base familiar desenvolvida para avaliar as condições de saúde bucal e a utilização de serviços no Brasil. A amostragem seguiu um desenho probabilístico complexo e multiestágio. Municípios foram selecionados dentro de cada domínio geográfico, e setores censitários e domicílios foram selecionados aleatoriamente. Todos os indivíduos elegíveis dentro das faixas etárias pré-definidas de 15–19, 35–44 e 65–74 anos foram convidados a participar. Os dados foram coletados por examinadores e entrevistadores treinados e calibrados, de acordo com os procedimentos descritos no manual oficial da pesquisa^19,20^.

A amostra inicial foi composta por 25.372 participantes com 15 anos de idade ou mais, dos quais 21.385 foram incluídos nas análises após excluir indivíduos com dados faltantes na variável dependente ou em variáveis independentes-chave. Uma abordagem de análise de caso completo foi adotada para garantir consistência na modelagem multivariável. As principais exclusões foram devido à ausência de respostas em medidas autorreferidas e à cor da pele.

Os dados extraídos da SB Brasil 2023 foram os seguintes: sexo (masculino ou feminino), faixa etária (15–19 anos, 35–44 anos e 65–74 anos), cor da pele autorreferida (branca, preta e parda), anos de escolaridade (sem estudo, 1–4 anos, 5–8 anos e 9 anos ou mais), autoavaliação da saúde bucal (muito boa, boa, razoável, ruim e muito ruim), necessidade percebida de tratamento (sim ou não), dor de dente autorreferida (sim ou não), dor facial autorreferida (sim ou não), impacto da saúde bucal na qualidade de vida (sim ou não) e localização do serviço odontológico utilizado (setor público ou privado).

De acordo com o Modelo Comportamental de Andersen, as variáveis independentes foram conceitualmente organizadas em três domínios. Os fatores predisponentes incluíram sexo, faixa etária, cor da pele autorreferida e anos de escolaridade. Os facilitadores incluíram tipo de serviço odontológico utilizado, cobertura municipal de saúde bucal na Atenção Básica e a disponibilidade de Centros de Especialidades Odontológicas (CEOs) no município. A necessidade percebida compreendeu a autoavaliação da saúde oral, a percepção da necessidade de tratamento, dor de dente autorreferida, dor facial autorreferida e o impacto da saúde bucal nas atividades diárias.

A cor da pele foi autorreferida pelo participante no momento da entrevista. Os participantes que não informaram a cor da pele, assim como os que relataram a cor da pele amarela ou indígena, foram excluídos da amostra devido à baixa frequência de casos. O impacto da saúde bucal na qualidade de vida foi avaliado pelo questionário *Oral Impacts on Daily Performances* (OIDP), composto por nove questões sobre a percepção de como a saúde bucal influencia as atividades da vida diária. Considerou-se que os participantes que responderam "não" a todas as perguntas não tiveram quaisquer impactos na qualidade de vida relacionada à saúde bucal. Dor de dente e dor facial autorreferidas referiram-se à presença de sintomas nos seis meses anteriores à entrevista, de acordo com o questionário da pesquisa.

Além das variáveis de nível individual, foram coletados dados contextuais municipais sobre a cobertura de saúde bucal na Atenção Básica e a disponibilidade de CEOs no município. Os CEOs são centros públicos de cuidados secundários dentro do SUS que oferecem procedimentos odontológicos especializados, incluindo endodontia, periodontia, cirurgia bucal e atendimento a pacientes com necessidades especiais. A cobertura municipal de saúde bucal na Atenção Básica foi definida como a proporção da população coberta pelas Equipes de Saúde Bucal dentro da Estratégia Saúde da Família, e foi obtida a partir de dados administrativos oficiais disponíveis na plataforma e-Gestor Atenção Primária à Saúde para o ano de 2023. A cobertura foi categorizada em até 80% e acima de 80%. Os dados sobre a disponibilidade de serviços de saúde foram extraídos de relatórios públicos na plataforma e-Gestor Atenção Primária à Saúde (https://relatorioaps.saude.gov.br/) para o ano de 2023.

A variável dependente foi a utilização recente de serviços odontológicos, operacionalizada como o tempo decorrido desde a última consulta odontológica, categorizado como até um ano e mais de um ano. Os indivíduos que relataram nunca ter visitado um dentista foram excluídos da análise.

Os dados foram importados e analisados usando o software Stata (versão 19). As estimativas ponderadas foram obtidas com base no desenho complexo do estudo, de acordo com as diretrizes da base de dados fornecida pelo Ministério da Saúde^20^.

Modelos de regressão logística multinível com intercepto aleatório no nível do município foram ajustados. Os efeitos fixos foram introduzidos de acordo com os domínios conceituais do Modelo de Andersen, incluindo fatores predisponentes, facilitadores, e necessidade percebida, como descrito anteriormente.

Dessa forma, um modelo hierárquico multinível foi construído para estimar o efeito cumulativo de determinantes individuais e contextuais na utilização recente de serviços odontológicos. O modelo conceitual adotado neste estudo, baseado no Modelo Comportamental de Andersen, e a organização das variáveis individuais e contextuais incluídas na análise são ilustrados na Figura 1.

**Figura 1:** Modelo conceitual de fatores associados à utilização recente de serviços odontológicos de acordo com o Modelo Comportamental de Andersen, SB Brasil 2023.

O ajuste do modelo foi avaliado utilizando o Critério de Informação de Akaike (*Akaike Information Criterion* – AIC) e o Critério de Informação Bayesiano (*Bayesian Information Criterion* – BIC), com valores mais baixos indicando melhor ajuste. A proporção de variância atribuível ao nível municipal foi estimada pelo Coeficiente de Correlação Intraclasse (*Intraclass correlation coefficient* – ICC). O modelo final, que considerou os efeitos das variáveis sociodemográficas, autorreferidas e relacionadas ao serviço, apresentou um ajuste significativamente bom.

O efeito das variáveis independentes foi estimado pela razão de chances (*odds ratio* – OR) utilizando um estimador robusto. As variáveis com valor p<0,05 foram consideradas estatisticamente significativas, considerando um intervalo de confiança de 95% (IC95%).

### Declaração de disponibilidade de dados:

Todo o conjunto de dados que dá suporte aos resultados deste estudo está disponível mediante solicitação ao autor correspondente. O conjunto de dados não está publicamente disponível devido à integração de bancos de dados realizada pelos autores.

## RESULTADOS

A amostra do estudo foi composta por 25.372 participantes. Deste total, 21.385 observações foram analisadas, uma vez que alguns participantes tinham dados faltantes. As frequências ponderadas das variáveis do estudo estão apresentadas na Tabela 1.

**Tabela 1:** Frequências ponderadas das variáveis estudadas e intervalo de confiança de 95%, considerando o desenho amostral complexo do estudo.

		Frequência ponderada (%)	IC95%	
Há quanto tempo você utiliza os serviços odontológicos?				
	Há aproximadamente 1 ano	51,89	48,99	54,76
	Há mais de um ano	48,11	45,24	51,01
Sexo				
	Masculino	38,76	37,13	40,42
	Feminino	61,24	59,58	62,87
Faixa etária (anos)				
	15 a 19	23,59	21,91	25,36
	35 a 44	55,60	53,37	57,80
	65 a 74	20,81	19,23	22,49
Cor da pele				
	Branca	44,63	41,03	48,28
	Preta	12,64	10,99	14,49
	Parda	42,73	39,35	46,19
Escolaridade (anos de estudo)				
	Sem estudo	2,95	2,40	3,62
	1 a 4	8,15	6,88	9,62
	5 a 8	14,71	13,19	16,38
	9 anos ou mais	74,19	71,52	76,69
Autoavaliação da saúde bucal				
	Muito boa	8,12	7,09	9,29
	Boa	46,01	43,81	48,23
	Razoável	33,43	31,59	35,32
	Ruim	9,47	8,15	10,96
	Muito ruim	2,98	2,29	3,86
Necessidade percebida de tratamento				
	Não	31,35	28,41	34,44
	Sim	68,65	65,56	71,59
Sente dor de dente				
	Não	79,19	77,05	81,17
	Sim	20,81	18,83	22,95
Sente dor facial				
	Não	83,15	81,03	85,07
	Sim	16,85	14,93	18,97
Impacto da saúde bucal na qualidade de vida				
	Sem impactos	51,27	47,77	54,77
	Com impactos	48,73	45,23	52,23
Localização do serviço odontológico utilizado				
	Setor público	51,09	47,62	54,55
	Setor privado	48,91	45,45	52,38
Cobertura de Saúde Bucal na APS				
	Até 80%	71,81	65,99	76,97
	Mais de 80%	28,19	23,03	34,01
Tem CEO				
	Não	28,64	20,89	37,88
	Sim	71,36	62,12	79,11

IC: Intervalo de confiança; APS: Atenção Primária à Saúde; CEO: Centro de Especialidades Odontológicas.

Do total de indivíduos incluídos na análise, 48,11% (IC95% 45,24–51,01) relataram a utilização de serviço odontológico há mais de um ano. Os participantes eram majoritariamente mulheres (61,24%; IC95% 59,58–62,87), com idades entre 35 e 44 anos (55,60%; IC95% 53,37–57,80) e autodeclarados como brancos (44,63%; IC95% 41,03–48,28), com nove ou mais anos de escolaridade (74,19%; IC95% 71,52–76,69). A autoavaliação da saúde bucal da maioria dos participantes foi classificada como "boa" (46,01%; IC95% 43,81–48,23) e 68,65% (IC95% 65,56–71,59) relataram necessidade percebida de tratamento odontológico. Dor de dente (20,81%; IC95% 18,83–22,95) e dor facial (16,85%; IC95% 14,93–18,97) foram menos frequentes, e a porcentagem de indivíduos que relataram impactos da saúde bucal na sua qualidade de vida foi equilibrada. Os participantes relataram utilizar serviços públicos em 51,09% (IC95% 47,62–54,55) dos casos, residir em cidades com cobertura de saúde bucal abaixo de 80% e com a presença de um CEO.

Os resultados de regressão logística multinível são apresentados na Tabela 2. Modelos sequenciais foram ajustados de acordo com o modelo conceitual de Andersen. No Modelo 1 (Fatores predisponentes), que incluiu apenas variáveis sociodemográficas, idade mais avançada e menor escolaridade estiveram fortemente associadas à utilização tardia de serviços odontológicos. Os indivíduos com idades entre 65 e 74 anos tinham probabilidades significativamente maiores de terem tido a última consulta odontológica há mais de um ano (OR 1,716; IC95% 1,477–1,995). Observamos um gradiente educacional evidente, com indivíduos com nove ou mais anos de escolaridade apresentando chances substancialmente menores de utilização tardia de serviços odontológicos (OR 0,381; IC95% 0,319–0,455). Indivíduos negros e pardos também apresentaram maiores probabilidades em comparação com os brancos. Após a inclusão das variáveis de necessidade reportadas pelos pacientes no Modelo 2, algumas associações foram atenuadas, mas permaneceram estatisticamente significativas, indicando mediação parcial das condições de saúde bucal percebidas. As associações mais fortes do modelo foram verificadas na autoavaliação da saúde bucal. Os indivíduos que autoavaliaram a sua saúde bucal como "muito ruim" apresentaram probabilidades consideravelmente maiores de utilização tardia de serviços (OR 2,821; IC95% 2,277–3,494). A necessidade percebida de tratamento permaneceu associada (OR 1,206; IC95% 1,084–1,341). Por outro lado, os participantes que relataram dor de dente tinham menos probabilidade de ter consultas odontológicas tardias (OR 0,419; IC95% 0,386–0,454), sugerindo a utilização de serviços orientada por sintomas. No modelo totalmente ajustado (Modelo 3), que incorporou facilitadores, a maioria das variáveis sociodemográficas e de necessidade permaneceram associadas à utilização tardia. Mulheres foram associadas a menores probabilidades de consultas odontológicas tardias em comparação com os homens (OR 0,799; IC95% 0,753–0,848). Indivíduos com idades entre 65 e 74 anos continuaram a apresentar probabilidades elevadas (OR 1,436; IC95% 1,242–1,660), embora com atenuação em comparação com o Modelo 1. Disparidades raciais persistiram, com indivíduos pretos (OR 1,161; IC95% 1,011–1,334) e pardos (OR 1,096; IC95% 1,005–1,196) apresentando maiores probabilidades de utilização tardia. A escolaridade manteve-se fortemente associada. Os participantes com nove ou mais anos de escolaridade tiveram uma probabilidade substancialmente menor de consultas odontológicas tardias (OR 0,383; IC95% 0,318–0,461), reforçando a magnitude do gradiente educacional.

**Tabela 2:** Modelo de Regressão Logística Multinível que estimou os fatores associados à utilização de serviços odontológicos há mais de um ano, considerando um modelo hierárquico de variáveis relacionadas a fatores socioeconômicos, medidas reportadas pelo paciente e disponibilidade de serviços.

		Modelo 1 (Variáveis sociodemográficas individuais)			Modelo 2 (Variáveis sociodemográficas+ PROM)			Modelo 3 (Variáveis sociodemográficas+ PROM+Serviços)		
		OR	IC95%		OR	IC95%		OR	IC95%	
Sexo (masculino)		ref.			ref.			ref.		
	Feminino	0,778	0,735	0,823	0,792	0,747	0,839	0,799	0,753	0,848
Faixa etária (15 a 19 anos)		ref.			ref.			ref.		
	35 a 44 anos	1,191	1,063	1,336	1,099	0,985	1,226	1,115	0,998	1,245
	65 a 74 anos	1,716	1,477	1,995	1,399	1,212	1,615	1,436	1,242	1,660
Cor da pele (branca)		ref.			ref.			ref.		
	Preta	1,217	1,075	1,378	1,193	1,045	1,362	1,161	1,011	1,334
	Parda	1,122	1,025	1,229	1,105	1,013	1,205	1,096	1,005	1,196
Escolaridade (sem estudo)		ref.			ref.			ref.		
	1 a 4 anos	0,797	0,649	0,980	0,799	0,643	0,992	0,824	0,657	1,032
	5 a 8 anos	0,633	0,533	0,751	0,620	0,521	0,737	0,631	0,527	0,756
	9 anos ou mais	0,381	0,319	0,455	0,367	0,307	0,440	0,383	0,318	0,461
Autoavaliação da saúde bucal (muito boa)					ref.			ref.		
	Boa				1,267	1,123	1,429	1,250	1,105	1,415
	Razoável				1,810	1,562	2,098	1,769	1,523	2,055
	Ruim				2,552	2,116	3,079	2,486	2,041	3,028
	Muito ruim				2,821	2,277	3,494	2,739	2,201	3,408
Necessidade percebida de tratamento (não)					ref.			ref.		
	Sim				1,206	1,084	1,341	1,202	1,079	1,338
Dor de dente (não)					ref.			ref.		
	Sim				0,419	0,386	0,454	0,413	0,382	0,448
Dor facial (não)					ref.			ref.		
	Sim				0,936	0,842	1,040	0,939	0,843	1,045
Impacto da saúde bucal na qualidade de vida (não)					ref.			ref.		
	Sim				0,829	0,754	0,912	0,830	0,754	0,914
Tipo de serviço utilizado (público)								ref.		
	Privado							0,820	0,732	0,919
Cobertura de saúde bucal na APS (até 80%)								ref.		
	Acima de 80%							0,904	0,750	1,090
Presença de CEO (não)								ref.		
	Sim							1,084	0,891	1,320
	**Modelo nulo**	**Modelo 1**			**Modelo 2**			**Modelo 3**		
ICC	9,05%	8,74%			8,07%			8,20%		
AIC	33580,53	31124,33			28018,19			27502,59		
BIC	33596,75	31205,07			28138,01			27646,06		

PROM: Medidas de Desfecho Reportados pelo Paciente (*Patient-Reported Outcome Measures*); OR: razão de chances (*odds ratio*); IC: Intervalo de confiança; ref.: Categoria de referência; APS: Atenção Primária à Saúde; ICC: Coeficiente de correlação intraclasse; AIC: Critério de Informação da Akaike; BIC: Critério de Informação Bayesiano.

Os maiores tamanhos de efeito continuaram a ser encontrados na necessidade percebida. Os indivíduos que autoavaliaram a sua saúde bucal como "muito ruim" tiveram três vezes mais chances de utilização tardia (OR 2,739; IC95% 2,201–3,408), representando a associação mais forte observada no modelo. A necessidade percebida de tratamento permaneceu associada (OR 1,202; IC95% 1,079–1,338). A dor de dente permaneceu inversamente associada (OR 0,413; IC95% 0,382–0,448).

Em relação aos facilitadores, a utilização de serviços odontológicos no setor privado esteve associada a menores probabilidades de utilização tardia (OR 0,820; IC95% 0,732–0,919). No entanto, a cobertura municipal de saúde bucal acima de 80% (OR 0,904; IC95% 0,750–1,090) e a presença de um CEO (OR 1,084; IC95% 0,891–1,320) não foram significativamente associadas.

Além disso, o ICC diminuiu de 9,05% no modelo nulo para 8,20% no modelo totalmente ajustado, indicando uma variabilidade contextual modesta, mas persistente entre os municípios.

## DISCUSSÃO

De acordo com os resultados deste estudo, as caraterísticas sociodemográficas e as medidas autorreferidas relacionadas à necessidade estão fortemente associadas à recente utilização de serviços odontológicos no Brasil. Embora a cobertura municipal de saúde bucal e a disponibilidade de CEOs não tenham sido estatisticamente associadas com o tempo desde a última consulta odontológica, os indivíduos que relataram utilizar serviços do setor privado tiveram maior probabilidade de ter tido uma consulta odontológica no ano anterior.

Resultados semelhantes foram observados em estudos nacionais^7,8,21^ cujos autores também identificaram desigualdades no uso de serviços odontológicos de acordo com o tipo de serviço, com uma vantagem consistente para os usuários do setor privado. Segundo tais estudos, ter um plano de saúde está associado a uma maior frequência de consultas odontológicas e uso regular de serviços, enquanto o acesso complementar permanece concentrado entre indivíduos com maior renda e escolaridade^8,21^. Além disso, os usuários do sistema público têm menor probabilidade de utilizar serviços odontológicos regularmente e apresentam maior tempo desde a última consulta, destacando a persistência de desigualdades estruturais na utilização de serviços de saúde bucal no Brasil^7^.

Nossos achados reforçam que fatores sociodemográficos, como cor da pele, escolaridade e idade, continuam sendo determinantes significativos da recente utilização de serviços odontológicos no Brasil, em consonância com estudos anteriores^5–7,13^. Observamos um evidente gradiente educacional, com maior escolaridade mostrando uma forte associação protetora contra consultas odontológicas tardias, sugerindo padrões sociais persistentes na utilização de serviços de saúde bucal.

Os fatores relacionados com a necessidade demonstraram as associações mais fortes no modelo totalmente ajustado. Os indivíduos que classificaram a sua saúde bucal como ruim ou muito ruim apresentaram uma probabilidade substancialmente maior de relatar consulta odontológica realizada há mais de um ano, enquanto os que relataram dor de dente apresentaram maior probabilidade de consulta odontológica recente, indicando padrões de utilização motivados por sintomas. Tais achados estão alinhados com a literatura, destacando o papel central da necessidade percebida no uso de serviços de saúde^1,2,13^, e reforçam a capacidade explicativa do modelo de Andersen quando aplicado a contextos de saúde bucal.

As fortes associações observadas para as medidas autorreferidas, em especial a autoavaliação da saúde bucal e a necessidade percebida de tratamento, sugerem que os indicadores relatados pelos pacientes estão intimamente relacionados com os padrões recentes de utilização de serviços odontológicos. Embora estratégias de triagem não tenham sido avaliadas neste estudo, esses achados indicam que medidas autorreferidas podem servir como marcadores relevantes para a identificação de grupos com diferentes perfis de utilização.

Neste contexto, autores de estudos recentes^22–24^ têm destacado o potencial das tecnologias digitais e da telemedicina na expansão do alcance dos serviços, na melhoria da qualidade da assistência e no acompanhamento das necessidades percebidas, especialmente no âmbito do SUS. Portanto, pesquisas futuras poderiam explorar como os indicadores relatados pelos pacientes podem ser integrados no planejamento do cuidado e nas estratégias de organização dos serviços, especialmente para populações socialmente vulneráveis.

Em relação aos facilitadores, não observamos uma associação estatisticamente significativa entre a cobertura municipal de saúde bucal ou a presença de um CEO e a utilização recente. Em certa medida, a expansão de serviços odontológicos no Brasil nos últimos 20 anos^25–28^ tem contribuído para reduzir as desigualdades a este respeito. No entanto, esses indicadores administrativos podem não captar totalmente dimensões como a acessibilidade geográfica, a organização de serviços, os tempos de espera ou a disponibilidade efetiva dos serviços. Dessa forma, a ausência de associação deve ser interpretada com cautela e não implica necessariamente que os fatores contextuais sejam irrelevantes, mas sim que as medidas disponíveis podem não abranger todas as dimensões relevantes dos recursos facilitadores.

Curiosamente, mesmo considerando apenas indivíduos que relataram utilizar serviços do setor público, não observamos efeito da cobertura de saúde bucal ou da presença de um CEO (dados não apresentados). Isso sugere que os indicadores estruturais de serviços, por si só, podem não explicar as diferenças nos intervalos de utilização, e podem refletir que as caraterísticas individuais e a necessidade percebida continuam a ser determinantes centrais^1,2,5,7,13^.

É importante ressaltar que este estudo avaliou o tempo desde a última consulta odontológica entre indivíduos que tinham acesso aos serviços anteriormente. Portanto, os achados não devem ser interpretados como medidas diretas de barreiras de acesso ou dificuldade em obter assistência. As diferenças observadas podem refletir variações na necessidade, preferências e crenças de saúde, organização de serviços ou modelos de cuidado, e os mecanismos causais não podem ser inferidos a partir do desenho transversal.

Como limitações, este estudo não considerou o efeito da variável "renda" na construção de modelos preditivos. Isto porque a variável "renda" tem uma frequência de não resposta de aproximadamente 30%, o que nos levou a omitir essa variável no estudo para evitar perder a robustez da análise da amostra. De qualquer modo, sabemos que a renda é um importante determinante social da utilização de serviços odontológicos e que a sua ausência pode limitar parcialmente a compreensão das desigualdades econômicas associadas ao acesso.

Além disso, o desfecho foi baseado em informações autorreferidas que podem estar sujeitas ao viés de memória. A natureza transversal do estudo impede a inferência temporal. Os indivíduos que nunca tinham visitado um dentista foram excluídos da análise, o que limita conclusões sobre o acesso absoluto aos serviços. Por fim, indicadores contextuais como a cobertura da Atenção Básica e a presença de CEO representam medidas administrativas e podem não refletir totalmente a disponibilidade, qualidade ou acessibilidade dos serviços em nível local.

Levando esses achados em consideração, os resultados reforçam a importância de abordagens que considerem a interação entre determinantes individuais, sociais e estruturais da utilização de serviços odontológicos. Quanto aos fatores predisponentes, a persistência de desigualdades sociais e raciais é consistente com a literatura, com ênfase na relevância de estratégias intersetoriais que visem reduzir as desigualdades e fortalecer a educação em saúde bucal^2,29^.

No que se refere aos facilitadores, fortalecer a Atenção Primária à Saúde Bucal e a integração entre as equipes de Saúde Bucal e os CEOs na rede de saúde são medidas essenciais para superar a fragmentação do cuidado, promover a continuidade entre os níveis de atenção e tornar o sistema mais eficaz e equitativo, reduzindo as desigualdades regionais^30^.

Por último, em relação à necessidade percebida, a incorporação de indicadores autorreferidos, como Medidas de Desfecho Reportados pelo Paciente (*Patient-Reported Outcome Measures*) e Medidas de Experiência Relatadas pelo Paciente (*Patient-Reported Experience Measures*), tem sido sugerida como uma estratégia para melhorar o monitoramento das necessidades e a avaliação da qualidade, apoiando abordagens com maior foco no paciente^31,32^.
